# Bone metabolism and inflammatory biomarkers in radiographic and non-radiographic axial spondyloarthritis patients: a comprehensive evaluation

**DOI:** 10.3389/fendo.2024.1227196

**Published:** 2024-02-15

**Authors:** Ignacio Gómez-García, Maria L. Ladehesa-Pineda, Juan M. Diaz-Tocados, Clementina López-Medina, Maria C. Abalos-Aguilera, Desiree Ruiz-Vilches, Guillermo Paz-Lopez, Andres Gonzalez-Jimenez, Juan A. G. Ranea, Alejandro Escudero-Contreras, Isabel Moreno-Indias, Francisco J. Tinahones, Eduardo Collantes-Estévez, Patricia Ruiz-Limón

**Affiliations:** ^1^ Department of Rheumatology, Reina Sofia University Hospital, Córdoba, Spain; ^2^ Maimonides Institute for Biomedical Research of Córdoba (IMIBIC), Cordoba, Spain; ^3^ Department of Medical and Surgical Sciences, University of Cordoba, Cordoba, Spain; ^4^ Vascular and Renal Translational Research Group, Biomedical Research Institute of Lleida, Dr. Pifarré Foundation (IRBLleida), Lleida, Spain; ^5^ Department of Molecular Biology and Biochemistry, Faculty of Science, University of Málaga, Málaga, Spain; ^6^ Bioinformatic Platform, The Biomedical Research Institute of Malaga and Platform in Nanomedicine (IBIMA-BIONANDPlatform), Malaga, Spain; ^7^ Centro de Investigación Biomédica en Red de Enfermedades Raras (CIBERER), Carlos III Health Institute, Madrid, Spain; ^8^ Spanish National Bioinformatics Institute (INB/ELIXIR-ES), Barcelona, Spain; ^9^ The Biomedical Research Institute of Malaga and Platform in Nanomedicine (IBIMA BIONAND Platform), Malaga, Spain; ^10^ Department of Endocrinology and Nutrition, Virgen de la Victoria University Hospital, Malaga, Spain; ^11^ Center for Biomedical Network Research (CIBER) in Physiopathology of Obesity and Nutrition (CIBEROBN), Carlos III Health Institute, Madrid, Spain; ^12^ Department of Medicine and Dermatology, Faculty of Medicine, University of Malaga, Malaga, Spain

**Keywords:** radiographic axial spondyloarthritis, bone metabolism, inflammation, biomarkers, Interleukin 13, sclerostin

## Abstract

**Introduction:**

Axial spondyloarthritis (axSpA) is a heterogeneous disease that can be represented by radiographic axSpA (r-axSpA) and non-radiographic axSpA (nr-axSpA). This study aimed to evaluate the relationship between the markers of inflammation and bone turnover in r-axSpA patients and nr-axSpA patients.

**Methods:**

A cross-sectional study included 29 r-axSpA patients, 10 nr-axSpA patients, and 20 controls matched for age and sex. Plasma markers related to bone remodeling such as human procollagen type 1 N-terminal propeptide (P1NP), sclerostin, tartrate-resistant acid phosphatase 5b (TRACP5b), receptor activator of nuclear factor kappa B ligand (RANKL), and osteoprotegerin (OPG) were measured by an ELISA kit. A panel of 92 inflammatory molecules was analyzed by proximity extension assay.

**Results:**

R-axSpA patients had decreased plasma levels of P1NP, a marker of bone formation, compared to controls. In addition, r-axSpA patients exhibited decreased plasma levels of sclerostin, an anti-anabolic bone hormone, which would not explain the co-existence of decreased plasma P1NP concentration; however, sclerostin levels could also be influenced by inflammatory processes. Plasma markers of osteoclast activity were similar in all groups. Regarding inflammation-related molecules, nr-axSpA patients showed increased levels of serum interleukin 13 (IL13) as compared with both r-axSpA patients and controls, which may participate in the prevention of inflammation. On the other hand, r-axSpA patients had higher levels of pro-inflammatory molecules compared to controls (i.e., IL6, Oncostatin M, and TNF receptor superfamily member 9). Correlation analysis showed that sclerostin was inversely associated with IL6 and Oncostatin M among others.

**Conclusion:**

Altogether, different inflammatory profiles may play a role in the development of the skeletal features in axSpA patients particularly related to decreased bone formation. The relationship between sclerostin and inflammation and the protective actions of IL13 could be of relevance in the axSpA pathology, which is a topic for further investigation.

## Introduction

Spondyloarthritis (SpA) is a heterogeneous group of chronic inflammatory rheumatic diseases that share common characteristics (1). This group includes several entities, such as the prototype axial spondyloarthritis (axSpA), which is the most frequent phenotype of axSpA, and the radiographic axial SpA (r-axSpA), which affects the axial skeleton and the sacroiliac joints with radiographic changes (1). On the other hand, the non-radiographic axSpA (nr-axSpA) includes patients with suggestive clinical characteristics of axSpA and the presence of bone marrow edema in the sacroiliac joint in magnetic resonance but no radiographic sacroiliitis (2).

In r-axSpA, the pathological process is characterized by disruption of the normal bone homeostasis, i.e., aberrant new bone formation and concomitant bone loss (3). Pathological bone formation, also known as osteoproliferation, produces structural damage to the spine in the form of syndesmophytes. As the disease progresses, these syndesmophytes can fuse the vertebrae, leading to a condition called ankylosis (4).

In contrast, excessive bone loss can result in osteopenia and osteoporosis, which can already be observed at the early stages of the disease. Severe loss of vertebral bone mineral density increased the risk of vertebral fractures, resulting in spinal deformities (5). Bone loss in r-axSpA may be influenced by several factors, including age, disease duration, gender, inflammation, new bone formation, diet, vitamin D levels, and immobility (6).

Inflammation is a key feature of r-axSpA, and it is thought to precede structural damage in affected joints. Therefore, several markers related to inflammation have been evaluated as prognostic biomarkers in axSpA. C-reactive protein (CRP) and erythrocyte sedimentation rate (ESR) are two commonly used markers of inflammation in clinical practice. High levels of CRP and ESR have been associated with increased disease activity and also with early radiographic progression in axSpA patients of the German Spondyloarthritis Inception Cohort (GESPIC) (7). Additionally, interleukin 6 (IL6) is a major pro-inflammatory cytokine that stimulates the formation and activity of bone-resorbing osteoclasts *in vitro* (8), playing a key role in bone disorders such as Paget´s disease (9).

Identifying biomarkers involved in bone metabolism and turnover could improve the management of r-axSpA. Previous studies conducted in axSpA have suggested that those patients who have syndesmophytes exhibited elevated levels of certain bone resorption markers, such as the C-terminal telopeptide fragments of type I collagen (CTX), as well as bone formation markers, such as osteocalcin (OC) and procollagen type I N-terminal peptide (P1NP) (3, 10). In a study conducted by Ruiz Heiland et al., lower serum levels of sclerostin and dickkopf-1 were found to be significantly associated with 2-year radiographic progression of the spine in patients with AS (11). On the other hand, higher serum levels of these Wnt pathway inhibitors were associated with no new syndesmophyte formation in AS (11, 12).

While there is a growing body of evidence indicating abnormal bone metabolism in patients with axSpA, the impact of inflammation on bone metabolism is still largely unknown. Therefore, this study aimed to evaluate the relationship between the markers of inflammation and bone turnover in r-axSpA patients and nr-axSpA patients.

## Materials and methods

### Population and study design

In this cross-sectional study, 29 r-axSpA patients, 10 nr-axSpA patients, and 20 controls matched for age and sex were included. Eligible patients had to fulfill the Assessment of SpondyloArthritis International Society (ASAS) classification criteria for the classification of axSpA (13). Patients who fulfilled the radiographic criteria established by the Modified New York Criteria were classified as r-axSpA, while those who did not fulfill these criteria were categorized as nr-axSpA (14). The patients belonged to the CASTRO cohort (Cordoba Ankylosing Spondylitis Task Registry and Outcomes) of the Reina Sofia University Hospital (Cordoba, Spain). Controls did not have a clinical history of axSpA or inflammatory diseases, or the presence of symptoms that could be related to an undiagnosed axSpA. The exclusion criteria were pregnancy, malignancies, chronic infections, other rheumatology diseases, extra-articular manifestations [psoriasis, inflammatory bowel disease (IBD), and uveitis], receiving biological disease-modifying antirheumatic drugs or treatment with drugs that could interfere with bone metabolism (bisphosphonates, strontium ranelate, selective estrogen receptor modulators, calcitonin, hormone therapy, denosumab, or teriparatide), and inability to understand the procedures to the protocol. All participants underwent a complete medical history physical examination and clinical chemistry analysis before enrolment. A case report form was used to collect the following clinical data: age, gender, disease duration, human leukocyte antigen (HLA)-B27 status, and current medications.

Disease activity was evaluated by the Bath Ankylosing Spondylitis Disease Activity Index (BASDAI) (15), Ankylosing Spondylitis Disease Activity Score (ASDAS; calculated with the CRP) (16), CRP (mg/L), and ESR (mm/h). Spinal mobility and functionality of patients were measured by the Bath Ankylosing Spondylitis Metrology Index (BASMI) (17) and Bath Ankylosing Spondylitis Functionality Index (BASFI) (15), respectively. Structural damage was assessed by the modified stoke Ankylosing Spondylitis Spine Score (mSASSS), which was evaluated by two trained readers (18). Bone mineral density (BMD) was evaluated by dual x-ray absorptiometry (DXA) BMD measurement of the total hip, lumbar, and femoral neck using a DXA LUNAR DPX 8548 BX-1 L densitometer (coefficient of variation < 1%). Exercise was measured using a questionnaire.

All participants provided written informed consent before being included. The study was conducted according to the principles of the Declaration of Helsinki, and the protocol was approved by the Ethics Committee of the Reina Sofía University Hospital, Córdoba, Spain.

### Blood sample collection and assessment of biochemical parameters

Peripheral venous blood samples were collected and processed as previously described (19). Plasma and serum were aliquoted and stored at −80°C for subsequent analysis. Laboratory markers of inflammation (ESR and CRP), the genetic factor human leukocyte antigen (HLA)-B27, lipid profile [total cholesterol, high-density lipoprotein (HDL), low-density lipoprotein (LDL), and triglycerides], serum glucose, ferritin, calcium, phosphate, 25-hydroxyvitamin D^3^ (25OH vit D), and parathyroid hormone (PTH) were quantified as part of routine patient management.

### Markers related to bone remodeling

To estimate the osteoblastic activity, also referred to as bone formation, human procollagen type 1 N-terminal propeptide (P1NP) quantification was carried out in plasma samples (1:200 dilution) using a commercial ELISA Kit (Cusabio Technology LLC, Houston, TX, USA) following the manufacturer’s instructions. In addition, the plasma levels of sclerostin, an inhibitor of bone formation that blocks the Wnt/β-catenin pathway (20), were also measured with a commercial ELISA kit (Cusabio Technology LLC) as indicated by the manufacturer.

The osteoclastic activity was determined by measuring plasma levels of tartrate-resistant acid phosphatase 5b (TRACP5b) and receptor activator of nuclear factor kappa B ligand (RANKL), both biomarkers of bone resorption. Plasma TRACP5b concentration was determined using ELISA kit (MyBioSource, San Diego, CA, USA) according to the manufacturer’s instructions. Circulating levels of RANKL were determined in plasma, diluted at 1:5, and then the quantification was performed with a commercial ELISA kit (Cusabio Technology LLC) as indicated by the manufacturer’s instructions. Additionally, the plasma concentration of osteoprotegerin (OPG), an inhibitor of RANKL that prevents osteoclast activity (21), was measured with an ELISA kit (RayBiotech, Peachtree Corners, GA, USA).

### Inflammation-related proteins measurement

A panel of 92 inflammatory mediators (Olink target 96 inflammation panel) was evaluated in the serum of all the participants included in the study using the highly specific and sensitive technology proximity extension assay (PEA) (Cobiomic Bioscience, Cordoba, Spain). PEA utilizes a dual-recognition immunoassay in which two matched antibodies labeled with distinct DNA oligonucleotides bind to a target protein in solution. This results in the proximity of the two antibodies, allowing their DNA oligonucleotides to hybridize. Following that, the hybridized oligonucleotides serve as a template for a DNA polymerase-dependent extension step, leading to the creation of a double-stranded DNA “barcode” unique to the specific antigen and quantitatively proportional to the initial concentration of the target protein. The hybridization and extension steps are followed by PCR amplification.

### Statistical analysis

A descriptive analysis of the variables was performed and expressed as percentages or as mean ± standard deviation (SD) or median [interquartile range (IQR)], as applicable. The normality of the distribution was assessed using the Kolmogorov–Smirnov test. Multiple comparisons were analyzed by one-way analysis of variance (ANOVA) with the Tukey test as *post-hoc* analysis for continuous data or by the Kruskal−Wallis test with Dunn multiple‐comparison test for non-normally distributed data. Categorical data were analyzed using Pearson’s chi-square test. Student’s *t*-test or Mann–Whitney *U* test was used to calculate differences between groups for numerical variables, and Pearson’s chi-square test was used for categorical variables. Differential protein analysis was performed using R programming language. Data normality and homoscedasticity were checked using base statistical tests from R. Shapiro–Wilk test was used to check the normality of the distribution of values of each molecule per group. Likewise, Levene’s test was used to assess homoscedasticity. The analysis of variance (ANOVA) test was applied to each molecule to detect differential abundance between groups. *Post-hoc* pairwise *t*-tests were applied subsequently on each pair of groups to check differences. Afterward, *p*-values were corrected using the FDR method to reduce the probability of observing type I errors, and fold change was calculated for each molecule. The sensitivity and specificity were evaluated for the most significant biomarkers using Receiver Operating Characteristic (ROC) curves that determine the sensitivity, specificity, and cutoff values. The effect sizes were analyzed using Cohen’s *d* test. Finally, volcano plots were made using the EnhancedVolcano package (version 1.12.0). The correlations were assessed by Spearman’s rank correlation. Molecules with at least one significative correlation value, depending on the p-value, were selected. Heatmaps were made using the pheatmap package (version 1.0.12). Statistical significance was set at *p* < 0.05. Statistical analyses and graph editions were performed with SPSS (15.0 version for Windows: SPSS, Chicago, IL, USA), GraphPad Prism 8.0.2 (GraphPad Software Boston, MA, USA), and R (version 4.1.3).

## Results

### Characteristics of the study population

**Table 1** shows the baseline characteristics of r-axSpA and nr-axSpA patients, and controls. Patients with r-axSpA had a larger disease duration compared to nr-axSpA patients (*p* = 0.024). Moreover, compared with the nr-axSpA and control groups, r-axSpA patients had higher levels of serum CRP (*p* = 0.003) and a greater frequency of arterial hypertension (*p* = 0.001). Furthermore, six r-axSpA patients were diagnosed with hyperlipidemia (*p* = 0.002) and received treatment to control lipid levels; therefore, lipid profiles were similar across the three groups. Regarding the BMD values, nr-axSpA patients displayed a significant decrease in the total hip BMD and femoral neck BMD compared to r-axSpA patients (*p* = 0.010 and *p* = 0.012, respectively). Moreover, compared to controls, r-axSpA patients showed a significant decrease in the femoral neck BMD (*p* = 0.046). No differences were found in terms of disease activity (evaluated with BASDAI and ASDAS), spinal mobility and functionality (measured by BASFI and BASMI), structural damage (mSASSS), HLA-B27 antigen, or treatments received [neither non-steroidal anti-inflammatory drugs (NSAIDs) nor sulfasalazine]. Plasma levels of calcium, phosphate, PTH, and 25-OH-VitD remained similar among all groups. The exercise was similar in these three groups.

**Table 1:** Clinical and laboratory parameters of all participants.

Variable	Controls (*n* = 20)	nr-axSpA (*n* = 10)	r-axSpA (*n* = 29)	*p*-value
Clinical parameters				
Age, years, mean (SD)	47.35 ± 10.98	38.10 ± 10.06	46.93 ± 13.09	0.104
Female sex, *n* (%)	8 (40)	2 (20)	13 (44.82)	0.379
Smoking				0.055
Never smoked, *n* (%)	9 (45)	4 (40)	9 (31.03)	
Exsmoker, *n* (%)	5 (25)	4 (40)	8 (27.58)	
Active smoker, *n* (%)	0 (0)	2 (20)	12 (41.37)	
Arterial hypertension, *n* (%)	2 (10)	0 (0)	6 (20.68) ^a,b^	0.001
Hyperlipidemia, *n* (%)	1 (5)	1 (10)	6 (20.68) ^a^	0.002
Exercise				0.553
Never or hardly ever, *n* (%)		3 (30)	14/24 (58.3)	
1–4 times/week, *n* (%)		5 (50)	8/23 (33.3)	
>4 times/week, *n* (%)		2 (20)	2/24 (8.3)	
BASDAI, median (IQR)	–	2.30 (1.45–5.30)	3.80 (2.00–4.65)	0.530
ASDAS, mean ± SD	–	2.09 ± 0.77	2.43 ± 0.96	0.334
BASMI, mean ± SD	–	2.18 ± 1.08	3.22 ± 1.83	0.102
BASFI, median (IQR)	–	1.90 (0.20–4.17)	3.40 (1.50–5.50)	0.281
mSASSS, median (IQR)	–	5.50 (2.75–8.25)	9.00 (3.00–22.00)	0.100
Total hip BMD, g/cm^2^, mean ± SD	1.02 ± 0.15	0.88 ± 0.12	1.03 ± 0.11^b^	0.032
Femoral neck BMD, g/cm^2^, mean ± SD	1.01 ± 0.13	0.86 ± 0.09	0.98 ± 0.12 a^,b^	0.017
Lumbar BMD, g/cm^2^, mean ± SD	1.11 ± 0.14	1.06 ± 0.17	1.11 ± 0.17	0.475
Disease duration, years, median (IQR)	–	5.00 (2.75–17.25)	19.00 (8.00–31.00)	0.024
Laboratory parameters				
HLA-B27, *n* (%)	–	8 (80)	22 (75.86)	0.652
CRP, mg/dL, median (IQR)	0.58 (0.50–3.47)	1.46 (0.12–5.14)	5.81 (2.85–10.19) ^a^	0.003
ESR, mm/h, median (IQR)	5.00 (3.00–10.00)	4.00 (4.00–6.00)	4.00 (3.50–6.50)	0.743
Total cholesterol, mg/dL, mean ± SD	197.66 ± 24.37	185.55 ± 25.76	185.73 ± 27.72	0.498
LDL, mg/dL, mean ± SD	119.11 ± 25.81	111.00 ± 32.93	110.00 ± 25.26	0.690
HDL, mg/dL, mean ± SD	54.66 ± 20.06	54.22 ± 19.38	57.30 ± 11.73	0.843
Tryglicerides, mg/dL, median (IQR)	109.00 (64.00–173.00)	91.00 (61.50–146.50)	79.00 (59.00–101.00)	0.409
Glucose, mg/dL, mean ± SD	81.88 ± 6.37	86.66 ± 5.93	82.40 ± 13.06	0.551
Ferritin, ng/mL, mean ± SD	118.57 ± 111.74	130.88 ± 71.75	81.60 ± 69.62	0.264
Corrected calcium for total protein, mg/dL, median (IQR)	9.50 (9.20–9.80)	9.20 (9.10–9.40)	9.30 (9.20–9.40)	0.127
Phosphate, mg/dL, median (IQR)	3.10 (2.70–3.15)	2.70 (2.50–3.25)	3.10 (2.70–3.80)	0.312
25OH vit D, ng/mL, median (IQR)	11.33 (9.43–16.60)	15.91 (13.58–18.58)	15.47 (11.60–20.70)	0.399
PTH, pg/mL, median (IQR)	40.50 (38.35–57.85)	48.20 (37.85–77.40)	38.20 (22.90–64.80)	0.361
Treatments				
NSAIDs (%)	–	8 (80)	24 (82.75)	0.764
Sulfasalazine (%)	–	0 (0)	2 (6.89)	0.394

ASDAS, Ankylosing Spondylitis Disease Activity Score; BASDAI, Bath Ankylosing Spondylitis Disease Activity Index; BASFI, Bath Ankylosing Spondylitis Functionality Index; BASMI, Bath Ankylosing Spondylitis Metrology Index; BMD, bone mineral density; CRP, C-reactive protein; ESR, erythrocyte sedimentation rate; HDL, high-density lipoprotein; HLA, human leukocyte antigen; IQR, interquartile range; LDL, low-density lipoprotein; mSASSS, modified Stoke Ankylosing Spondylitis Spine Score; nr-axSpA, non-radiographic axial spondyloarthritis; NSAIDs, non-steroidal anti-inflammatory drugs; PTH, parathyroid hormone; r-axSpA, radiographic axial spondyloarthritis; SD, standard deviation; 25OH vit D, 25-hydroxyvitamin D^3^. Differences between groups; ^a^p < 0.05 vs. control; ^b^p < 0.05 vs. nr-axSpA.

### Plasma biomarkers of bone turnover in radiographic and non-radiographic axial SpA patients

**Figure 1:**
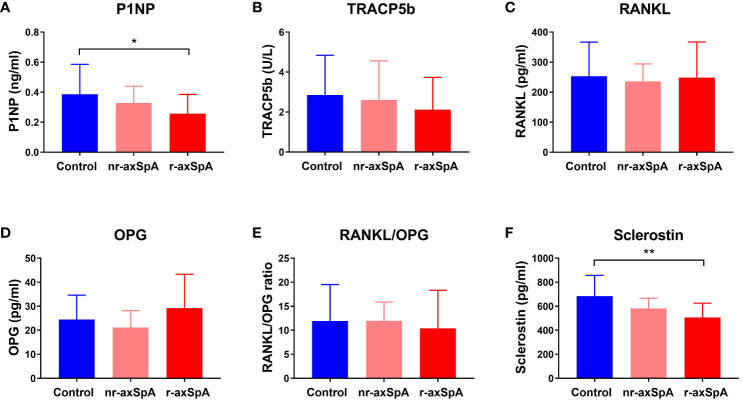
Plasma bone markers in radiographic and non-radiographic axial SpA patients, and controls. The circulating levels of **(A)** procollagen type I N-terminal propeptide (P1NP), indicative of osteoblast activity, **(B)** tartrate-resistant acid phosphatase 5b (TRACP5b), and **(C)** receptor activator of nuclear factor kappa-B ligand (RANKL), both markers of osteoclast activity, were measured. Additionally, levels of **(D)** osteoprotegerin (OPG), a RANKL decoy receptor that inhibits osteoclast function, **(E)** RANKL/OPG ratio, and **(F)** Sclerostin, an inhibitor of Wnt signaling that modulates bone formation, were also analyzed. Bars represent mean ± standard deviation. Significant differences: *p < 0.05; **p < 0.01.

To investigate whether bone turnover was altered differently in both axSpA patient groups and controls, we measured the levels of well-known bone biomarkers in plasma samples. We found that the circulating levels of the P1NP were significantly decreased (effect size, 0.376) in r-axSpA patients compared to the control group (**Figure 1A**), indicating lower bone formation; however, no significant differences were observed in the plasma levels of TRACP5b and RANKL, both markers of osteoclast activity (**Figures 1B, C**, respectively). We also measured OPG plasma concentration (**Figure 1D**), a decoy receptor that binds to RANKL and prevents interaction with its receptor RANK, and similarly, we did not observe significant differences among the three groups, consistently with similar levels of the RANKL/OPG ratio (**Figure 1E**). Interestingly, we found a significant reduction in the plasma concentration of sclerostin in r-axSpA patients as compared with controls (**Figure 1F**), which was not associated with the decrease in plasma P1NP concentration, suggesting that other factors would be involved.

### Analysis of inflammatory-related proteins in patients and controls

**Figure 2:**
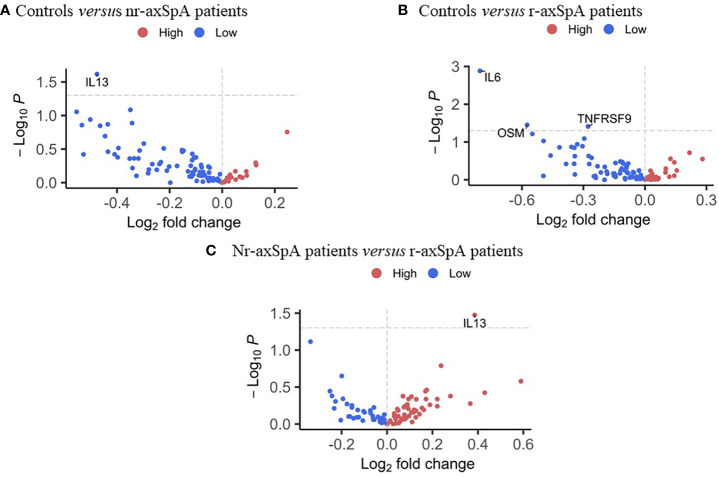
Differential protein profile between radiographic and non-radiographic axial SpA patients, and controls. **(A)** Volcano plot of 92 inflammation-related proteins in controls compared to non-radiographic axial SpA patients. **(B)** Volcano plot of 92 inflammation-related proteins in controls compared to radiographic axial SpA patients. **(C)** Volcano plot of 92 inflammation-related proteins in non-radiographic axial SpA patients compared to radiographic axial SpA patients.

The levels of 92 serum inflammatory-related proteins were evaluated in this study. Nr-axSpA patients exhibited significantly increased IL13 levels compared to controls and r-axSpA patients with effect sizes of 0.371 and 0.646, respectively (**Figures 2A, C**). Compared to r-axSpA patients, controls exhibited significantly decreased levels of IL6, Oncostatin M (OSM), and TNF-receptor named tumor necrosis factor receptor superfamily member 9 (TNFRSF9), with effect sizes of 0.911, 0.629, and 0.266, respectively (**Figure 2B**).

**Figure 3:**
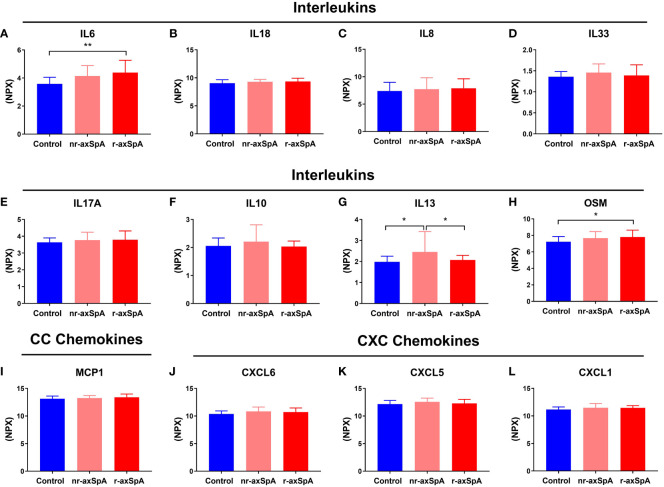
Proteins associated with inflammation in patients with radiographic and non-radiographic axial spondyloarthritis (axial SpA) and controls. The proteins were categorized based on their functions into different groups: Interleukins, which includes Interleukin 6 (IL6), IL18, IL8, IL33, IL17A, IL10, IL13 and Oncostatin M (OSM), (**A–H**, respectively); CC chemokines **(I)**, including Monocyte Chemoattractant Protein-1 (MCP1); and CXC chemokines, which includes C-X-C Motif Chemokine Ligand 6 (CXCL6), CXCL5 and CXCL1 (**J–L** respectively). The bars depict the mean ± standard deviation of normalized protein expression units (NPX) values. Significant differences: *p < 0.05 and **p < 0.01.

Inflammatory-related proteins were divided into five groups based on their functions: CC Chemokines, CXC Chemokines, interleukins (ILs), cell surface molecules and receptors, and other cytokines and proteins. Notably, patients with r-axSpA showed elevated plasma levels of IL6 compared to the control group (4.38±0.876 vs 3.58±0.469, *p* = 0.003), while nr-axSpA patients did not exhibit a significant increase (**Figure 3A**). Plasma levels of IL18, IL8, IL33, IL17-A and IL10 remained similar among all groups (**Figures 3B–F**). Interestingly, nr-axSpA patients showed increased levels of IL13 compared to controls (2.45±0.968 vs 1.97±0.274, *p* = 0.024) and r-axSpA patients (2.45±0.968 vs 2.07±0.219, *p* = 0.033; **Figure 3G**). Furthermore, Oncostatin M (OSM) plasma levels were also slight but significantly increased in r-axSpA patients compared to controls (control 7.80±0.828 vs 7.23±0.627, p=0.012; **Figure 3H**). No differences were observed in the plasma levels of Monocyte Chemoattractant Protein-1 (MCP-1), CXC Motif Chemokine Ligand 6 (CXCL6), CXCL5 and CXCL1 among groups (**Figures 3I–L** respectively).

**Figure 4:**
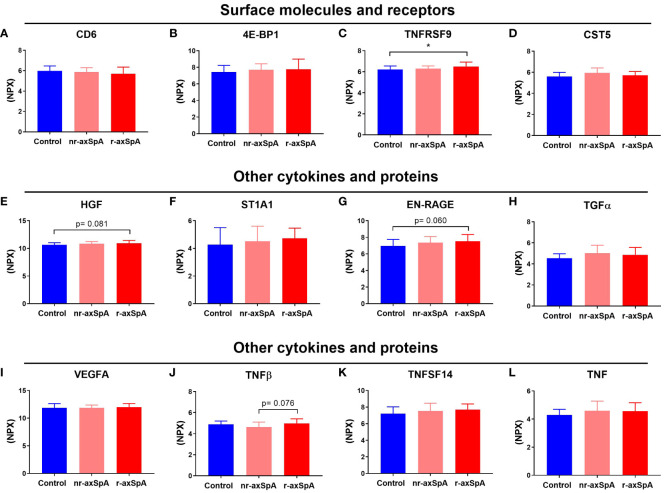
Proteins related to inflammation in axial spondyloarthritis (axial SpA) patients, both with and without radiographic changes, and controls. The proteins were categorized into different groups based on their functions: cell surface molecules and receptors, including Cluster of Differentiation 6 (CD6), Eukaryotic initiation factor 4E-binding protein 1 (4E-BP1), Tumor Necrosis Factor Receptor Superfamily Member 9 (TNFRSF9), and Cystatin D (CST5), (**A–D** respectively); or other cytokines and proteins, including Hepatocyte growth factor (HGF), Sulfotransferase 1A1 (ST1A1), Extracellular Newly identified Receptor for Advanced Glycation End-products binding protein (EN-RAGE), Transforming Growth Factor Alpha (TGFα), Vascular Endothelial Growth Factor A (VEGFA), Tumor Necrosis Factor β (TNFβ), Tumor Necrosis Factor Superfamily Member 14 (TNFSF14), and Tumor Necrosis Factor TNF, (**E–L**, respectively). The bars indicate the mean ± standard deviation of normalized protein expression units (NPX) values. Significant differences: *p < 0.05.

Regarding the cell surface molecules and receptors involved in inflammatory processes, the plasma levels of the Cluster of Differentiation 6 (CD6) and Eukaryotic initiation factor 4E-binding protein 1 (4E-BP1) were similar among all groups (**Figures 4A, B** respectively). However, patients with r-axSpA exhibited a slight but significant increase in the plasma levels of TNFRSF9 compared to controls (6.50±0.420 vs 6.21±0.330, *p* = 0.014; **Figure 4C**). No differences were observed in the circulating concentration of Cystatin-D (CST5, **Figure 4D**). Respect to other cytokines and proteins related to inflammation evaluated in this study, we observed that plasma levels of Hepatocyte Growth Factor (HGF) showed a tendency to increase in r-axSpA patients as compared to control group (*p* = 0.081, **Figure 4E**). Sulfotransferase 1A1 (ST1A1) plasma levels were similar among groups (**Figure 4F**), while the Extracellular Newly identified Receptor for Advanced Glycation End-products binding protein (EN-RAGE) also showed a tendency to increase in r-axSpA patients compared to the control group (*p* = 0.060, **Figure 4G**). Plasma levels of Transforming growth factor alpha (TGFα) and Vascular Endothelial Growth Factor A (VEGFA) remained similar in all groups (**Figures 4H, I** respectively). Of note, r-axSpA patients showed a tendency to increase the plasma levels of tumor necrosis factor β (TNFβ) compared to those with nr-axSpA (*p* = 0.076, **Figure 4J**). The circulating levels of Tumor Necrosis Factor Superfamily Member 14 (TNFSF14) and TNF were similar in all groups (**Figures 4K, L** respectively).

### Analysis of inflammatory-related proteins and the bone turnover markers as potential biomarkers of disease in axSpA

**Figure 5:**
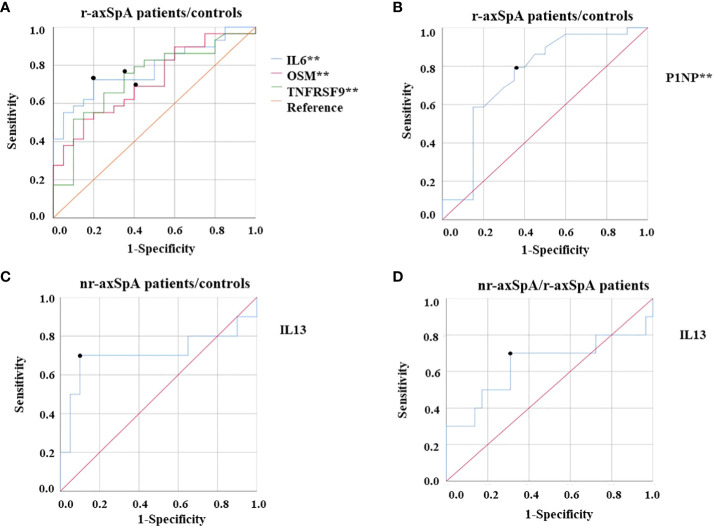
ROC curve analyses of circulating levels of inflammatory-related proteins and bone turnover markers as potential biomarkers of disease in axSpA. **(A)** Positive relation of inflammatory-related proteins in r-axSpA patients compared with controls [IL6 (AUC 0.78; *p* = 0.001), OSM (AUC 0.72; *p* = 0.011), and TNFRSF9 (AUC 0.73; *p* = 0.006)]. **(B)** Negative relation in P1NP in r-axSpA patients compared to controls (AUC 0.75; *p* = 0.003). **(C)** Positive relation in IL13 in nr-axSpA patients compared to controls (AUC 0.71; *p* = 0.065). **(D)** Positive relation in IL13 in nr-axSpA patients compared to r-axSpA patients (AUC 0.63; *p* = 0.198). Significant differences **p < 0.01.

To further investigate the potential utility of the inflammatory-related proteins and the bone turnover markers in terms of differentiating the two pathological conditions in axSpA, the sensitivity (S) and specificity (E) of the most relevant molecules in this study were evaluated using the ROC curve. A positive significant relation was found for IL6 (AUC 0.78, 95% CI 0.66–0.91, *p* = 0.001; S 72%; E 80%), OSM (AUC 0.72, 95% CI 0.57–0.86, *p* = 0.011; S 69%; E 60%), and TNFRSF9 serum levels (AUC 0.73, 95% CI 0.57–0.86, *p* = 0.006; S 76%; E 65%) in r-axSpA patients compared to controls (**Figure 5A**). In addition, a negative significant relation was observed for P1NP plasma levels (AUC 0.75, 95% CI 0.60–0.90, *p* = 0.003; S 79%; E 65%) in r-axSpA patients compared to controls (**Figure 5B**). Finally, there was a positive but not significant association in serum IL13 levels in nr-axSpA patients compared to controls (AUC 0.71, 95% CI 0.47–0.95, *p* = 0.065; S 76%; E 65%; **Figure 5C**) and in nr-axSpA patients compared to r-axSpA (AUC 0.63, 95% CI 0.40–0.88, *p* = 0.198; S 70%; E 69%; **Figure 5D**).

### Correlation studies between inflammatory-related proteins, bone turnover markers, and bone mineral density

**Figure 6:**
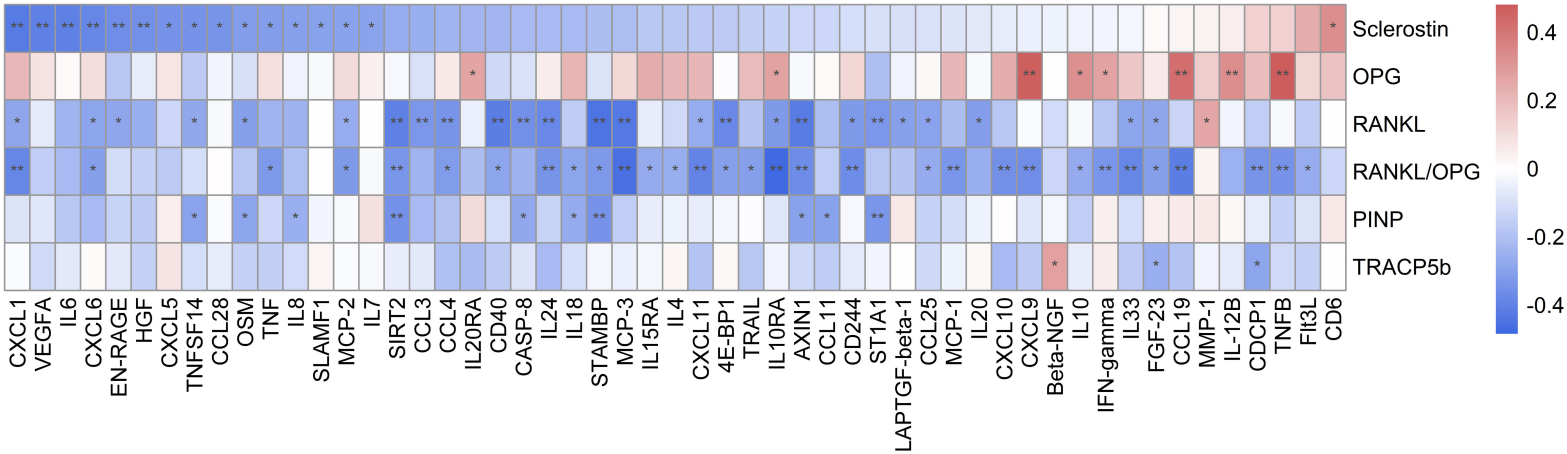
Heatmap representing correlations between bone remodeling markers and inflammatory-related proteins. Significant differences: *p ≤ 0.05 and **p ≤ 0.01.

Results from correlation analysis revealed several significant associations between inflammatory proteins, bone turnover markers, and bone mineral density. Specifically, we found that plasma sclerostin concentration was inversely correlated with interleukins (IL6, IL8, IL7, and OSM), CC chemokines (CCL28 and MCP-2), CXC chemokines (CXCL1, CXCL5, and CXCL6), and other cytokines and proteins (VEGFA, SLAMF1, HGF, TNF, EN-RAGE, and TNFSF14), and a positive correlation with surface proteins and receptors (CD6). On the other hand, P1NP was inversely correlated with interleukins (IL8, OSM, and IL18), CC chemokines (CCL11), and other cytokines and proteins (AXIN1, SIRT2, CASP-8, ST1A1, STAMBP, and TNFSF14) (**Figure 6**). These findings provide further evidence of the crosstalk between bone metabolism and inflammation in the axSpA pathophysiology. Concerning BMD test, lumbar BMD was inversely associated with interleukins (IL1α, IL2, IL20, IL33, and IL5). Femoral neck BMD was inversely correlated with interleukins (IL12B), CXC chemokines (CXCL9 and CX3CL1), surface molecules and receptors (IL22RA1), and other cytokines and proteins [colony-stimulating factor 1 (CSF1), caspase 8 (CASP8), artemin (ARTN), and fibroblast growth factor 5 (FGF5)]. Finally, total hip BMD was also inversely correlated with surface molecules and receptors (IL10RA, IL15RA, IL10RB, and IL22RA1), CXC chemokines (CX3CL1), and other cytokines and proteins [thymic stromal lymphopoietin (TSLP), stem cell factor (SCF), FGF5, beta nerve growth factor (BETA-NGF), ARTN, FMS-like tyrosine kinase 3 ligand (Flt3l), and CASP8] (**Supplementary Table 1**).

## Discussion

Our data showed differential inflammatory status and bone remodeling in axSpA patients, as reflected by an augmentation of serum pro-inflammatory cytokines and a decrease in bone formation in r-axSpA patients. Consequently, the inflammatory status of r-axSpA patients may result in altered bone turnover, resulting in decreased bone quality and an increased risk of fractures and deformities (22). These findings found in r-axSpA patients were not observed in nr-axSpA patients, who also did not show any differences in serum bone markers. Of note, nr-axSpA patients had higher levels of serum IL13, which could exert a preventive effect on inflammation (23). These findings may suggest that the differential inflammatory profile could be a primary cause of bone alterations.

We observed that the circulating levels of the P1NP, a bone formation marker (24), were decreased in r-axSpA as compared with the control group, indicating decreased osteoblast activity, whereas nr-axSpA remained similar. These results indicate that, at the systemic level, bone formation is reduced in r-axSpA despite the aberrant new bone formation, which may explain, at least in part, the osteopenia and osteoporosis conditions in these patients (25). No differences among groups were observed in the markers of osteoclast activity, TRACP5b (26) and RANKL (21), as well as in OPG plasma concentration, a decoy receptor for RANKL and inhibiting RANKL-RANK binding through it (27). Consistently, the RANKL/OPG ratio remained similar among the three groups, indicating similar osteoclast activity. As compared with the control group, we observed a significant reduction in plasma sclerostin concentration in r-axSpA patients. It is interesting to note that sclerostin is a well-known inhibitor of osteogenesis, blocking the activation of the canonical Wingless-related integration site (Wnt) pathway, which plays a key role in bone formation (28). Therefore, the lower levels of sclerostin in r-axSpA patients are not in line with the expected decrease in plasma P1NP concentration. However, previous studies have also observed lower levels of sclerostin in patients with r-axSpA, which has been linked to increased structural damage and disease progression in these patients (12, 29). In the same way, Luchetti MM et al. also observed low serum levels of sclerostin in patients with axSpA/IBD. These authors suggested that this reduction in sclerostin levels might contribute to the development of axial joint inflammation (30). Moreover, an association of serum Dickkopf-1(DKK1) levels has been reported, which is also an inhibitor of the Wnt pathway, with BMD values and the prevalence of vertebral fractures, suggesting that DKK1 could also contribute to bone abnormalities in individuals with axSpA (31). Additionally, sclerostin is also associated with other functions such as pro-inflammatory processes (32).

The underlying pathophysiology of axSpA is thought to be driven by inflammation, which results in systemic and local bone loss and local new bone formation (33). Inflammation in this pathology is mediated by pro-inflammatory cytokines, such as IL6, TNF-α, and IL17 (34, 35).

Our data demonstrated an inflammatory profile in r-axSpA, displayed by significantly high concentrations of pro-inflammatory markers in serum such as IL6, OSM, and TNFRSF9 (HGF, EN-RAGE, and TNFβ showed a tendency) as compared to nr-axSpA controls. Some factors could influence the inflammatory profile, such as smoking or exercise, but non-significant differences were observed in our study (36, 37).

Regarding IL6, which is a pleiotropic cytokine produced by various cell types in the context of infection, inflammation, and malignancy, our findings were in line with other works that found elevated IL6 levels in the serum of SpA patients (38, 39). Interestingly, other authors observed that Infliximab treatment decreases IL6 serum levels in patients with ankylosing spondylitis, and this depletion correlated with improvement in disease activity and bone mineral density (40).

In addition, elevated levels of OSM were observed in r-axSpA patients in this study. OSM is a cytokine that belongs to the IL6 family and plays a role in inflammation and bone remodeling. Tsui FWL et al. proposed that both Lipocalin 2 and OSM-associated pathways were involved in axSpA pathogenesis (41). Of note, it has been demonstrated that OSM inhibits sclerostin production in primary murine osteoblasts, also indicating a principal effect of inflammation in the regulation of bone markers (42), as may also occur in r-axSpA patients.

Another inflammatory-related marker increased in r-axSpA patients was TNFRSF9, which is involved in the modulation of inflammation and its dysregulation has been linked to various pathological conditions, including cancer, chronic viral infections, and autoimmune disorders. According to our results, a recent study has observed a high expression of *TNFRSF9* in the Treg cells of synovial fluid of SpA patients (43). Whether TNFRSF9 levels in plasma are related to higher *TNFRSF9* expression in the Treg cells must be clarified.

ROC curve analysis revealed that serum levels of IL6, OSM, and TNFRSF9 could be used as predictive biomarkers for radiological axSpA progression.

In addition, it has been reported that axSpA patients show high levels of IL17, which might increase bone resorption and compromise bone homeostasis (44). In fact, IL17 inhibitors have been suggested as potential treatments to prevent bone loss (45, 46). Nevertheless, other studies have shown that IL17 can promote increased osteoblastic differentiation, which could potentially contribute to excessive osteogenesis and bone formation (47, 48). This phenomenon may be, at least in part, attributed to the activation of the Janus Kinase (JAK) 2/STAT3 pathway (49). In our study, we did not observe any change regarding serum levels of IL17A and plasma levels of osteoclast activity markers such as RANKL or TRACP5b.

On the other hand, our nr-axSpA patients displayed increased levels of IL13 compared to controls and r-axSpA patients. This cytokine has presented contradictory results in rheumatoid arthritis (RA) patients. Some studies have found upregulated levels of IL13 in serum (50) and other studies did not differ in IL13 levels from the control group (51). IL13 is a component of Th-2-mediated immunity, and it has been proposed that IL13 can downregulate IL23 from antigen-presenting cells or IL17 from T cells, thus blocking IL17-driven inflammation (52). This finding could suggest that cytokines such as IL13, with anti-inflammatory properties, may have the potential to prevent bone alterations. However, further in-depth studies are necessary to clarify these findings. For example, fetuin-A, a mineral metabolism protein, has been proposed as a promising marker in axSpA severity. In this respect, Favero et al. (53) have suggested that fetuin-A levels could be a useful biomarker for identifying axSpA patients with a high risk of developing severe disease and early structural damage.

To additionally explore the relationship between inflammatory markers and bone remodeling markers, we next performed correlation studies and observed an inverse correlation between P1NP and sclerostin with pro-inflammatory markers. Specifically, we found that plasma levels of sclerostin were significantly inversely correlated with serum cytokine levels (IL6 and OSM). A study by Wehmeyer et al. (54) observed that the lack of sclerostin or its inhibition with neutralizing antibodies led to increased activation of pathways involved in inflammatory states in a mouse model of RA, indicating that sclerostin could have a protective effect against TNF-dependent inflammation. Conversely, another study reported that RA patients treated with anti-TNFα showed increased levels of sclerostin (55). A wide range of effects on bone turnover is likely to occur as a result of sclerostin modulation in inflammatory and non-inflammatory conditions. As an example, anti-TNFα agents were shown to increase serum levels of bone formation markers, such as P1NP, and suppress bone resorption markers in RA and SpA (56). These findings suggest that changes in bone biomarkers due to anti-inflammatory therapies are associated with improvements in the disease activity of RA and SpA. Therefore, it would be interesting to comprehensively study other therapies that act on different pathways of inflammation such as IL6, and observe if bone remodeling markers undergo changes that translate into an improvement in patients with r-axSpA.

Even though the number of participants collected in each group is one of the limitations of our study, these individuals were well-phenotyped and many variables have been collected. Additionally, another limitation of our study was the unavailability of trabecular bone score measurements. However, although further studies are required, our results reinforce what has been described in the literature about the relationship between inflammatory markers and bone turnover markers, which could lead to new insights into the pathogenesis of the disease and potential therapeutic targets.

## Conclusion

In summary, r-axSpA patients showed decreased bone formation as assessed by lower plasma levels of P1NP. In addition, these patients showed decreased sclerostin levels compared to controls, which were related to the plasma levels of inflammatory cytokines rather than bone formation markers in r-axSpA. Conversely, nr-axSpA patients demonstrated elevated levels of the anti-inflammatory cytokine IL13, suggesting potential protective effects. Further studies are required to elucidate the role of sclerostin in the inflammatory processes in r-axSpA patients and the potential protective actions of IL13 in those lacking radiographic features. While further investigation is required, these findings offer insights that may contribute to identifying novel targets and therapies for patients with axSpA in relation to bone alterations and inflammation.

## Data availability statement

The raw data supporting the conclusions of this article will be made available by the authors, without undue reservation.

## Ethics statement

The studies involving humans were approved by Ethics Committee of the Reina Sofía University Hospital, Córdoba, Spain. The studies were conducted in accordance with the local legislation and institutional requirements. The participants provided their written informed consent to participate in this study.

## Author contributions

IG-G, MLL-P, JMD-T, and CL-M wrote the first draft of the manuscript. IG-G, MLL-P, CL-M, MCA-A, DR-V, AE-C carried out patient recruitment and data collection. JMD-T and PR-L were major contributors to laboratory determinations and contributors to the interpretation of laboratory data. GP-L, AG-J, and JAGR performed statistical analyses. FJT, IM-I, reviewed and edited. PR-L, EC-E were contributors to the design of the study and interpretation of patient data, and major contributors in writing the manuscript. All authors had final approval of the submitted and published versions. All authors have read and agreed to the published version of the manuscript.
