# Supplementary material for: Bone metabolism and inflammatory biomarkers in radiographic and non-radiographic axial spondyloarthritis patients: a comprehensive evaluation
Source: Front Endocrinol (Lausanne). 2024 Feb 15;15:1227196. doi: 10.3389/fendo.2024.1227196 (PMC10915870; doi:10.3389/fendo.2024.1227196)
Supplement: Supplementary file 1 [file DataSheet_1.pdf]

Supplementary Table 1. Spearman´s rank correlations between inflammatory-related proteins and bone mineral density.

	**Lumbar BMD (g/cm^2)**	**Femoral neck BMD (g/cm^2)**	**Total hip BMD (g/cm^2)**
IL1α	*r*=-0.564 *p*=0.002		
IL2	*r*=-0.39 *p*=0.044		
IL12B		*r*=-0.387 *p*=0.034	
IL20	*r*=-0.392 *p*=0.042		
IL33	*r*=-0.53 *p*=0.004		
IL5	*r*=-0.442 *p*=0.021		
CXCL9		*r*=-0.402 *p*=0.028	
CX3CL1		*r*=-0.374 *p*=0.042	*r*=-0.414 *p*=0.026
IL10RA			*r*=-0.424 *p*=0.022
IL10RB			*r*=-0.401 *p*=0.031
IL15RA			*r*=-0.438 *p*=0.018
IL22RA1		*r*=-0.408 *p*=0.025	*r*=-0.469 *p*=0.010
FGF5		*r*=-0.554 *p*=0.001	*r*=-0.391 *p*=0.036
TSLP			*r*=-0.386 *p*=0.039
SCF			*r*=-0.431 *p*=0.019
BETA-NGF			*r*=-0.369 *p*=0.049
ARTN		*r*=-0.466 *p*=0.009	*r*=-0.389 *p*=0.037
Flt3l			*r*=-0.415 *p*=0.025
CASP8		*r*=-0.367 *p*=0.046	*r*=-0.373 *p*=0.047
CSF1		*r*=-0.407 *p*=0.026	

Abbreviations: ARTN, artemin; BETA-NGF, beta nerve growth factor; BMD, bone mineral density; CASP8, caspase 8; CSF1, colony-stimulating factor 1; CXCL9, C-X-C motif chemokine ligand 9; CX3CL1, C-X3-C motif chemokine ligand 1; FGF5, fibroblast growth factor 5; Flt3l,

FMS-like tyrosine kinase 3 ligand; SCF, stem cell factor; TSLP, thymic stromal lymphopoietin. *r* values of Spearman's rank correlation and *p* values of their null hypothesis are shown. The correlations shown here are only those that are significant (p=0.05).
